# *Ancyronyx lianlabangorum* sp. nov., a new spider riffle beetle from Sarawak, and new distribution records for *A. pulcherrimus* Kodada, Jäch & Čiampor based on DNA barcodes (Coleoptera, Elmidae)

**DOI:** 10.3897/zookeys.1003.55541

**Published:** 2020-12-14

**Authors:** Ján Kodada, Manfred A. Jäch, Hendrik Freitag, Zuzana Čiamporová-Zaťovičová, Katarína Goffová, Dávid Selnekovič, Fedor Čiampor Jr

**Affiliations:** 1 Department of Zoology, Faculty of Natural Sciences, Comenius University in Bratislava, Mlynská dolina B-1, SK-842 15, Bratislava, Slovakia Comenius University Bratislava Slovakia; 2 Naturhistorisches Museum Wien, Burgring 7, A–1010, Wien, Austria Naturhistorisches Museum Wien Vienna Austria; 3 Ateneo de Manila University, Biology Department, School of Science and Engineering, Loyola Heights, Quezon City, 1108, Philippines Ateneo de Manila University Quezon Philippines; 4 Universiti Brunei Darussalam, Environmental and Life Sciences, Faculty of Science, Jalan Tungku Link, Gadong, BE1410, Brunei Universiti Brunei Darussalam Gadong Brunei; 5 Museum für Naturkunde, Leibniz Institute for Evolution and Biodiversity Science, Invalidenstraße 43, Berlin, 10115, Germany Leibniz Institute for Evolution and Biodiversity Science Berlin Germany; 6 Zoology Lab, Plant Science and Biodiversity Centre, Slovak Academy of Sciences, Dúbravská cesta 9, SK-84523, Bratislava, Slovakia Plant Science and Biodiversity Centre, Slovak Academy of Sciences Bratislava Slovakia

**Keywords:** *
Ancyronyx
*, Coleoptera, Elmidae, taxonomy, variability

## Abstract

*Ancyronyx lianlabangorum***sp. nov.** (Coleoptera, Elmidae), a new spider riffle beetle from the Kelabit Highlands (Sarawak, northern Borneo), is described. Illustrations of the habitus and diagnostic characters of the new species and the similar, polymorphic *A. pulcherrimus* Kodada et al. are presented. Differences to closely related species, based on COI nucleotide sequences and morphological characters, are discussed. *Ancyronyx pulcherrimus* is here recorded from Sarawak for the first time, based on DNA barcoding.

## Introduction

Borneo, the third-largest island of the world, possesses a very diverse fauna with numerous endemic species including riffle beetles (e.g., Kodada et al. 2014, 2020; Freitag et al. 2018). Tragically, the primary forests of Borneo continually decrease together with their specific aquatic habitats, which are disappearing or deteriorating at an alarming rate (Faridah-Hanum et al. 2018). In the scope of our recent research on the diversity of Dryopidae and Elmidae (Insecta, Coleoptera) in Sarawak, we examined numerous streams in the Kelabit Highlands, Gunung Mulu National Park (NP), and in the Kuching Division. During several months of fieldwork in 2018 and 2019, we noticed that especially the sensitive cloud forest ecosystems are now drastically influenced by climate change. This phenomenon was extremely notable in the Kelabit Highlands and Gunung Mulu NP, with very unpredictable weather conditions, local floods and increased temperatures.

So far, seven species of *Ancyronyx* Erichson have been recorded from Borneo: *A. acaroides* Grouvelle (Brunei, Sabah, Sarawak); *A. clisteri* Kodada et al. (Brunei, Sabah, Sarawak); *A. malickyi* Jäch (Sabah, Sarawak); *A. procerus* Jäch (Brunei, Sabah, Sarawak); *A. pulcherrimus* Kodada et al. (Brunei); *A. reticulatus* Kodada et al. (Sabah); and *A. sarawacensis* Jäch (Brunei, Sabah, Sarawak). Interestingly, all these species belong to the *Ancyronyx variegatus* (Germar) species group, representing larger species inhabiting submerged wood (Freitag and Jäch 2007). Contrarily, in the neighboring islands of the Philippines and Sulawesi, the majority of the species belongs to the *A. patrolus* Freitag & Jäch species group, preferring rocky substrates (Freitag and Balke 2011; Freitag 2013; Freitag and Kodada 2017a, b).

The macropterous *Ancyronyx acaroides*, *A. malickyi*, and *A. procerus* are good flyers and considered to be widespread in Southeast Asia (Jäch et al. 2016); however, the taxonomic and distributional data have been confirmed at a molecular level for only *A. procerus* (Kodada et al. 2020). The remaining four species seem to have restricted ranges and are obviously endemic to Borneo. Fully developed long wings were found in several specimens of *A. sarawacensis* and *A. clisteri* .The remaining individuals studied are most probably macropterous too, considering the elytral shape.

During our fieldwork in the Kelabit Highlands we found a new, large and flightless species resembling *A. pulcherrimus* and *A. reticulatus*. Besides, we discovered populations of *A. pulcherrimus* in the Gunung Mulu NP and in the Kuching Division, which differ to some extent in coloration and morphological characters from the type specimens from Brunei.

Here, we present the description of the new species, *A. lianlabangorum* sp. nov. and provide an analysis of the COI variation along with the examination of the morphological variability in the *A. pulcherrimus* clade with an attempt to discuss possible taxonomic solutions.

## Material and methods

Most of the specimens examined were collected from numerous watercourses in Sarawak during three field trips in 2018 and 2019. Adults were collected individually from submerged wood and preserved in 96% ethanol, specifically for the use of DNA barcoding, and samples were as soon as possible stored at –22 °C.

The material used for the study is deposited in the following collections: **BM** (Brunei Museum, Brunei); **CFDS** (Forest Department Sarawak, Kuching, Malaysia); **CKB** (collection of Ján Kodada, Bratislava, Slovakia); **NMW** (Naturhistorisches Museum Wien, Austria); **UB** (Department of Biology, Universiti Brunei, Darussalam, Brunei); **UBDM** (Universiti Brunei Darussalam Museum, Brunei).

In addition to the fresh material, older dry-pinned specimens were soaked in warm water with several drops of concentrated acetic acid and cleaned. Abdomina with genitalia or genitalia only were placed in lactic acid for one or two days and temporarily mounted onto microscopic slides. Specimens were examined and measured using a Leica M205C stereomicroscope with fusion optics and diffuse lighting at magnifications up to 160×. For measurements, an eyepiece graticule (5 mm: 100) or the Leica MC190-HD camera attached to the microscope and LAS software were used. The specimens were photographed under a Zeiss Axio Zoom.V16 stereomicroscope using diffuse LED lighting and a Canon 5D Mark IV camera attached. Dissected genitalia and pregenital segments were studied and illustrated while mounted on a temporary microscope cavity slide covered with a cover glass at magnifications up to 640× with a Leica DM 1000 microscope using a Leica drawing device.

For examination of the wing polymorphism, one elytron was entirely removed, which, unfortunately, resulted often in breaking the elytron due to the firm interlocking devices. Because of the limited number of specimens available, we therefore dissected only a few specimens with different elytral shapes.

Principal component analyses (PCA) were performed separately for male and female specimens using software PAST 3.12 (Hammer et al. 2001) and a variance-covariance matrix with log-transformed variables. PCA plots were subsequently edited in Adobe Illustrator CC.

Metric characters of 13 males and nine females of *A. lianlabangorum* sp. nov. as well as 12 males and 16 females of *A. pulcherrimus* were used for the PCA analyses; all intact specimens identified by mtDNA characters are in the dataset of measurements. Morphometric parameters are provided in tables as range and mean ± standard deviation. The following characters were measured: **BL** (body length without head, length of pronotum and elytra measured along midline); **EL** (elytral length, measured along suture from level of the most anterior point to the most posterior tip in dorsal view); **EW** (maximum combined elytral width); **HW** (head width including eyes); **ID** (interocular distance); **MW** (maximum pronotal width); **PL** (pronotal length along midline).

DNA was extracted from 39 adults representing five species of *Ancyronyx* and three species of *Graphelmis*. Of these, 31 were sequenced successfully, and their datasets were submitted to GenBank (see accession numbers in Table 1). DNA was extracted from the whole specimen or one entire hind leg including the metacoxa with some muscles using E.Z.N.A. Tissue DNA kit (OMEGA bio-tek) or Qiagen Blood and Tissue kit according to the manufacturer’s protocols for DNA Extraction from Tissue. A fragment of the 5′ end of the mitochondrial gene for cytochrome c oxidase subunit I (COI) was amplified with primers LCO1490 and HCO2198 (Folmer et al. 1994). PCR was amplified according to the protocol on https://zsm-entomology.de/wiki/The_Beetle_D_N_A_Lab. Amplification products were mainly purified with Exo-CIP Rapid PCR Cleanup Kit (New England Biolabs), and both strands sequenced by the commercial service of Macrogen Europe Inc. (Amsterdam, Netherlands). Raw sequences were assembled and edited in Geneious v. 6.1.8 (https://www.geneious.com). The genetic distances and maximum likelihood (ML) tree were measured by the K2P model with bootstrap support (1,000 replicates) and performed in MEGA v. 7 software (Kumar et al. 2016). The best-fitted substitution model (GTR+I+G) was selected by jModelTest 2 (Darriba et al. 2012). The maximum likelihood tree based on amino acid sequences was measured by the JTT matrix-based model with bootstrap support (1,000 replicates) and performed in MEGA v. 7 software. Voucher IDs for sequenced specimens are provided between square brackets.

The general morphological terminology follows Kodada et al. (2016) and Lawrence and Ślipiński (2013).

**Table 1.** Samples used in the molecular analyses: origin of samples, GenBank accession numbers.

Specimens, voucher IDs	Origin	GenBank no.
*Ancyronyx lianlabangorum* JK147	Malaysia, Sarawak	MT367499
*Ancyronyx lianlabangorum* JK146	Malaysia, Sarawak	MT367500
*Ancyronyx lianlabangorum* JK144	Malaysia, Sarawak	MT367501
*Ancyronyx lianlabangorum* JK145	Malaysia, Sarawak	MT367502
*Ancyronyx pulcherrimus* FZ1632A	Malaysia, Sarawak	–
*Ancyronyx pulcherrimus* FR324	Brunei	MT568725
*Ancyronyx pulcherrimus* JK100	Malaysia, Sarawak	MT367503
*Ancyronyx pulcherrimus* JK101	Malaysia, Sarawak	MT367504
*Ancyronyx pulcherrimus* JK102	Malaysia, Sarawak	MT367505
*Ancyronyx pulcherrimus* JK214	Malaysia, Sarawak	MT367506
*Ancyronyx pulcherrimus* JK215	Malaysia, Sarawak	MT367507
*Ancyronyx pulcherrimus* JK213	Malaysia, Sarawak	MT367508
*Ancyronyx pulcherrimus* JK208	Malaysia, Sarawak	MT367509
*Ancyronyx pulcherrimus* JK195	Malaysia, Sarawak	MT367510
*Ancyronyx pulcherrimus* JK106	Malaysia, Sarawak	MT367511
*Ancyronyx pulcherrimus* JK193	Malaysia, Sarawak	MT367512
*Ancyronyx pulcherrimus* FR344	Malaysia, Sarawak	MT568724
*Ancyronyx pulcherrimus* JK12	Malaysia, Sarawak	MT367513
*Ancyronyx pulcherrimus* JK199	Malaysia, Sarawak	MT367514
*Ancyronyx pulcherrimus* JK105	Malaysia, Sarawak	MT367515
*Ancyronyx pulcherrimus* JK197	Malaysia, Sarawak	MT367516
*Ancyronyx procerus* JK143	Malaysia, Sarawak	MT367517
*Ancyronyx procerus* JK142	Malaysia, Sarawak	MT367518
*Ancyronyx procerus* JK37	Malaysia, Sarawak	MT367519
*Ancyronyx sarawacensis* JK38	Malaysia, Sarawak	MT367520
*Ancyronyx sarawacensis* JK39	Malaysia, Sarawak	MT367521
*Ancyronyx clisteri* H44	Brunei	LR735553
*Ancyronyx clisteri* FZ1640	Malaysia, Sarawak	MK505421
*Graphelmis monticola* FZ530	Malaysia, Kelantan	MK505416
*Graphelmis anulata* FZ510	Malaysia, Pahang	MK505424
*Graphelmis obesa* FZ544	Malaysia, Sabah	MK505408

## Results

### DNA analysis

**Figure 1.**
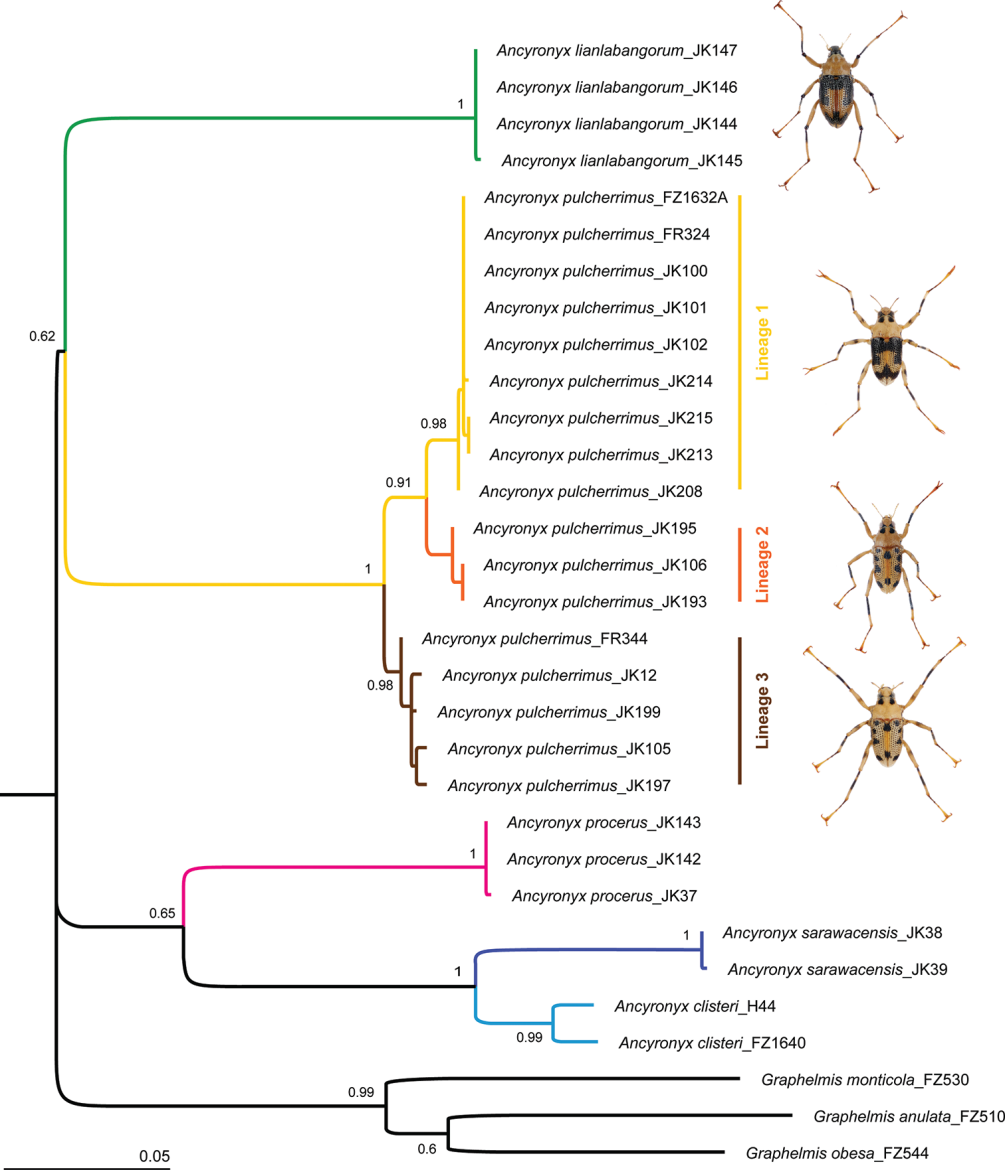
Maximum Likelihood tree inferred from aligned COI mtDNA nucleotides sequences.

**Figure 2.**
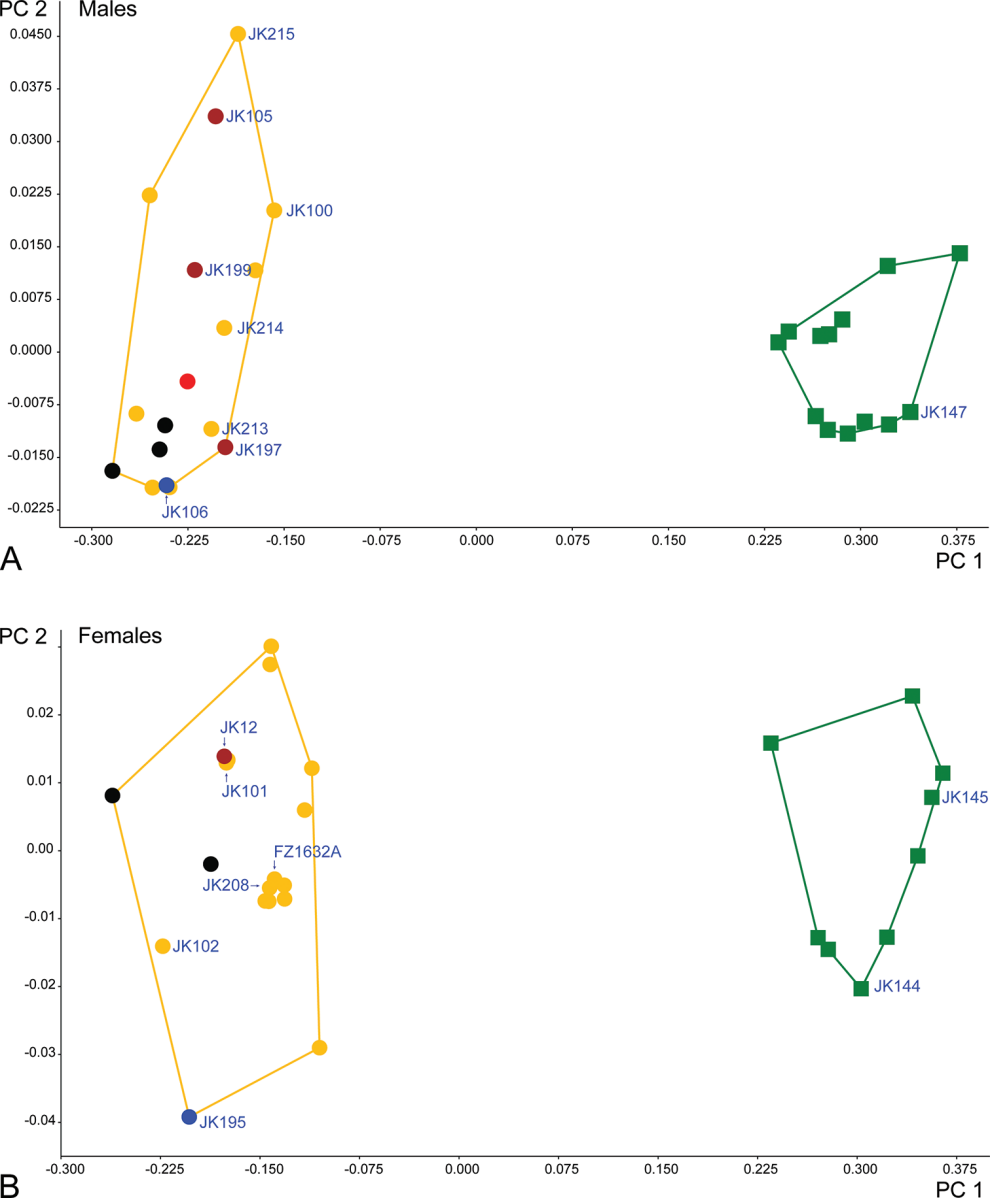
Results of the PCA analysis showing ordination of *Ancyronyx lianlabangorum* sp. nov. and *A. pulcherrimus* Kodada, Jäch & Čiampor specimens along the first two principal components **A** males **B** females. Clusters of *A. pulcherrimus* specimens on left side of plot (yellow lines), clusters of *A. lianlabangorum* sp. nov. on right side (green lines). Yellow circles: *A. pulcherrimus* specimens from Gunung Mulu NP, first lineage; blue circles: *A. pulcherrimus* from Bayur River, second lineage; brown circles: *A. pulcherrimus* from Bayur River, third lineage; black circles: *A. pulcherrimus* from Bayur River, specimens not barcoded, not assignable to the first or second lineage according to morphology; red circle: *A. pulcherrimus* from Brunei; green squares: *A. lianlabangorum* sp. nov. Genetic voucher IDs are included according to Table 1.

The COI sequences used in the analysis are 648 bp long, with no ambiguous sites or indels. The ML analysis revealed several well-separated and highly supported, monophyletic clades representing at least five *Ancyronyx* species (Fig. 1). The interspecific divergences were high and varied from 9.4% to 20.0% (Table 2, Suppl. material 1: Table S1). The intraspecific distance ranged from 0.0% to 0.2% in *Ancyronyx lianlabangorum* sp. nov.; it was 2.5% in *A. clisteri*, 0.2% in *A. sarawacensis*, and ranged from 0.0 to 0.2% in *A. procerus*. Mean genetic distances within species are shown in Table 2. More complex divergences for the two latter species were calculated on larger sample sizes from different populations studied by Kodada et al. (2020). Surprisingly, 17 new sequences obtained from two widely separated, allopatric populations of the well-supported *A. pulcherrimus* clade, showed higher pairwise genetic distances ranging from 0.0% to 3.7%. Specimens grouped in three lineages with high statistical support (Fig. 1), two of them appeared to be distinguishable by the elytral color pattern. The first lineage involves sequences of specimens from Gunung Mulu NP sampled from a stream ca 15 km from the type locality and of one specimen from Brunei. This dataset shows only a minimal nucleotide substitution pattern (Suppl. material 2: Table S2) and morphological characters correspond to those of the type specimens of *A. pulcherrimus*. Beyond any doubt, they are conspecific. The second and the third lineages are morphologically similar to each other. Still, they differ from the first lineage in the elytral color pattern, and their genetic distances differs from the first lineage in 1.7% to 3.2% respectively. Although the second and third lineages are sympatric, they differ in the pattern of nucleotide substitutions and their genetic divergence ranges from 2.5% to 3.7% (Suppl. material 1: Table S1). Contrarily, the examination of the external and genital morphology as well as the measurements of the specimens sequenced failed to provide characters useful to distinguish these sympatric lineages (Fig. 2, Table 4). Despite the relatively high divergences within the entire *A. pulcherrimus* clade based on the consensus nucleotide sequences, all specimens were grouped in a single well-supported and unstructured lineage in the tree inferred from amino acid sequences (Suppl. material 3: Figure S1, Suppl. material 4: Table S3). The very uniform genital morphology also confirms this status. Thus, we consider all specimens of this clade being conspecific.

**Table 2.** Pairwise genetic distances (*p*-distance) between five *Ancyronyx* species and the genus *Graphelmis* (outgroup) and mean interspecific genetic distance of five *Ancyronyx* species.

		1	2	3	4	5	within species (mean)
1	*A. lianlabangorum* sp. nov.						0.1 %
2	*A. pulcherrimus*	16.9 %					1.8 %
3	*A. procerus*	16.7 %	17.2 %				0.1 %
4	*A. sarawacensis*	19.6 %	19.0 %	17.8 %			0.2 %
5	*A. clisteri*	20.0 %	18.9 %	15.4 %	9.4 %		2.5 %
6	*Graphelmis* (outgroup)	20.0 %	20.0 %	19.1 %	20.7 %	20.7 %	

### PCA analysis

We performed PCA analyses based on seven characters to quantify and display morphometric variations among *Ancyronyx pulcherrimus* and *A. lianlabangorum* sp. nov. (Fig. 2, Table 4). The first principal component (PC 1) explained 98.87% of the variance in males and 98.45% in females (Table 3). According to the loadings (Table 3), it was strongly correlated with elytral length and width in both sexes. The second principal component (PC 2) explained 0.37% of the variance in males and 0.50% in females, and correlates most strongly with pronotal length and width in both sexes. The analysis revealed two clusters, each representing a different clade/species, distinctly separated along PC 1 axis which strongly correlates with the elytral length and width. The variance revealed by the PCA analysis corresponds to the differences in the actual measurements; body length, elytral length, and elytral width of *A. lianlabangorum* sp. nov. are distinctly greater than in *A. pulcherrimus* (Fig. 2, Table 4). PCA analyses did not confirm the three lineages detected by the phylogenetic analysis of COI nucleotide sequences within the *A. pulcherrimus* clade.

**Table 3.** Loadings onto the principal components for males and females of *Ancyronyx lianlabangorum* sp. nov. and *A. pulcherrimus*. The first and second highest values for each PC are highlighted in bold.

	Males			Females		
	PC 1	PC 2	PC3	PC 1	PC 2	PC3
Explained variance (%)	98.87	0.37	0.34	98.45	0.50	0.46
Loadings of variables:						
BL	0.381	-0.109	-0.270	0.380	0.191	-0.020
EL	**0.420**	-0.630	-0.2981	**0.410**	0.195	-0.327
EW	**0.435**	-0.063	-0.196	**0.445**	0.034	-0.419
PL	0.338	**0.657**	-0.240	0.330	**0.302**	**0.749**
MW	0.354	**0.273**	-0.012	0.342	**0.196**	**0.239**
HW	0.324	0.234	**0.268**	0.323	0.060	-0.244
ID	0.381	-0.160	**0.818**	0.398	-0.889	0.198

**Table 4.** Metric characters of *Ancyronyx lianlabangorum* sp. nov. and *A. pulcherrimus*, parameters are provided as range and mean ± standard deviation.

	*A. lianlabangorum* sp. nov.		*A. pulcherrimus*		*A. pulcherrimus*		*A. pulcherrimus*		*A. pulcherrimus*	
			Aggregated data		Lineage 1		Lineage 2		Lineage 3	
	Males	Females	Males	Females	Males	Females	Males	Females	Males	Females
	*N* = 13	*N* = 9	*N* = 17	*N* = 18	*N* = 9	*N* = 14	*N* = 1	*N* = 1	*N* = 3	*N* = 1
BL: mm	2.54–3.01 2.73 ± 0.12	2.67–3.01 2.86 ± 0.11	1.62–1.84 1.74 ± 0.06	1.70–2.04 1.89 ± 0.09	1.68–1.84 1.76 ± 0.06	1.76–2.04 1.92 ± 0.06	1.70	1.76	1.68–1.76	1.88
**EL: mm**	**1.86–2.08 1.95 ± 0.07**	**1.90–2.14 2.04 ± 0.08**	**1.12–1.24 1.19 ± 0.04**	**1.18–1.40 1.30 ± 0.06**	**1.12–1.24 1.93 ± 0.04**	**1.18–1.40 1.32 ± 0.05**	**1.20**	**1.20**	**1.14–1.24**	**1.30**
EW: mm	1.26–1.36 1.32 ± 0.04	1.30–1.42 1.37 ± 0.05	0.74–0.82 0.78 ± 0.03	0.78–0.88 0.84 ± 0.02	0.76–0.82 0.79 ± 0.02	0.80–0.88 0.84 ± 0.02	0.74	0.80	0.80–0.82	0.82
**BL/EW**	**2.02–2.21 2.08 ± 0.05**	**2.04–2.16 2.09 ± 0.03**	**2.10–2.42 2.23 ± 0.08**	**2.09–2.43 2.26 ± 0.07**	**2.17–2.42 2.25 ± 0.08**	**2.20–2.43 2.29 ± 0.06**	**2.30**	**2.20**	**2.10–2.15**	**2.29**
EL/EW	1.44–1.53 1.49 ± 0.02	0.93–1.02 0.97 ± 0.03	1.39–1.63 1.52 ± 0.07	1.47–1.67 1.56 ± 0.05	1.39–1.63 1.52 ± 0.07	1.48–1.67 1.57 ± 0.05	1.62	1.50	1.43–1.51	1.59
**PL: mm**	**0.78–0.90 0.82 ± 0.03**	**0.78–0.92 0.86 ± 0.05**	**0.50–0.60 0.55 ± 0.03**	**0.54–0.66 0.60 ± 0.03**	**0.52–0.60 0.56 ± 0.03**	**0.56–0.66 0.61 ± 0.03**	**0.52**	**1.58**	**0.54–0.56**	**0.60**
MW: mm	1.02–1.16 1.08 ± 0.04	1.00–1.20 1.11 ± 0.06	0.68–0.76 0.71 ± 0.03	0.70–0.84 0.77 ± 0.04	0.68–0.76 0.72 ± 0.03	0.72–0.84 0.78 ± 0.03	0.70	0.72	0.72–0.76	0.76
**PL/MW**	**0.70–0.78 0.75 ± 0.02**	**0.74–0.79 0.77 ± 0.02**	**0.74–0.82 0.77 ± 0.03**	**0.73–0.85 0.78 ± 0.03**	**0.74–0.83 0.78 ± 0.03**	**0.73–0.85 0.78 ± 0.03**	**0.74**	**0.81**	**0.74–0.78**	**0.79**
HW: mm	0.60–0.68 0.62 ± 0.02	0.62–0.68 0.65 ± 0.02	0.40–0.46 0.42 ± 0.02	0.42–0.48 0.46 ± 0.02	0.40–0.44 0.42 ± 0.02	0.44–0.48 0.46 ± 0.02	0.42	0.44	0.42–0.46	0.44
**ID: mm**	**0.34–0.40 0.37 ± 0.01**	**0.34–0.40 0.39 ± 0.02**	**0.22–0.26 0.23 ± 0.01**	**0.22–0.28 0.25 ± 0.01**	**0.22–0.26 0.24 ± 0.01**	**0.24–0.28 0.25 ± 0.01**	**0.24**	**0.26**	**0.22–0.24**	**0.24**

#### Ancyronyx lianlabangorum sp. nov.

Taxon classificationAnimaliaColeopteraElmidae

79F211AB-1C32-5F3E-9EC5-AB416A067D30

http://zoobank.org/FC6E0E5C-1FE9-431E-BB0B-DF81F5D44FB1

##### Type locality

**(Fig. 5A).** Pa’ Ramudu River (a tributary of Pa’ Kelapang River), 3°32'16.2"N, 115°30'22.5"E, ca 900 m a.s.l.; meandering, 7–15 m wide, shallow, slowly flowing through a degraded primary forest; environment of Kampung Ramudu, Kelabit Highlands, Sarawak, Malaysia.

##### Type material.

***Holotype*** ♂ [JK147] (CFDS): “Malaysia, Sarawak, Miri distr., Ramudu env., 5.03.2019, (No. 51), 3°32'16.2"N, 115°30'22.5"E, ca. 900 m a.s.l., Ramudu riv., J. Kodada & D. Selnekovič lgt.”. ***Paratypes*** (CFDS, CKB, NMW): 10 ♂♂ [incl. JK146], 4 ♀♀ [incl. JK144, JK145]: same label data as holotype; 3 ♂♂, 4 ♀♀: “Malaysia, Sarawak, Miri distr., Ramudu env., 5.–6.03.2019, (No.50), Pa’ Masia riv., 3°31'57.1"N, 115°30'41.4"E, ca. 970 m a.s.l., J. Kodada & D. Selnekovič lgt.”; 1 ♀: “Malaysia, Sarawak, Miri distr., Ramudu env., 28.06.2018, (18) 03°32'50.8"N, 115°29'25.9"E, 920 m a.s.l., Pa’ Kasi riv., J. Kodada & D. Selnekovič lgt.”.

##### Diagnosis.

*Ancyronyx lianlabangorum* sp. nov. represents one of the largest species characterized by: (1) large size: body length ca 2.5–3.0 mm and elytral width ca 1.3–1.4 mm; (2) comparatively short obovate body form; (3) shiny black head and unicolored yellow pronotum; (4) black-yellowish color pattern of elytra with moderately prevailing black; (5) femora yellowish around middle, lacking dark spot; (6) aedeagus large, with subparallel-sided, apically abruptly narrowed penis; parameres robust, shorter and reaching only up to apical third of penis length, apices wide and emarginate near middle; (7) ovipositor robust, with numerous conspicuous peg-like sensilla; stylus moderately long: ca 0.43× as long as distal portion of coxite; distal part of coxite short and wide: 1.16× as long as wide at middle; longitudinal baculum of paraprocts about twice as long as coxite.

The morphologically most similar *A. pulcherrimus* can be distinguished by: (1) distinctly smaller size: body length ≤2.0 mm and elytral width ≤0.9 mm; (2) subparallel-sided elongate body form; (3) bicolored head and pronotum; (4) black-yellowish color pattern of elytra with moderately prevailing yellow; (5) femora with dark spot near middle; (6) smaller aedeagus with sides of penis subparallel in basal half and gradually tapering in apical half; parameres long, nearly reaching tip of penis, apices subtruncate and wide; (7) ovipositor slender, with fewer conspicuous peg-like sensilla; distal portion of coxite longer and thinner: 1.8× as long as wide at middle; longitudinal baculum of paraprocts ca 1.6× as long as coxite; (8) about 17% divergence of the partial mtDNA for cytochrome c oxidase subunit COI.

*Ancyronyx reticulatus*, the second similar species, differs from the new species in: (1) distinctly smaller size: body length ≤2.1 mm and elytral width ≤0.9 mm; (2) subparallel-sided elongate body form; (3) bicolored head and pronotum; (4) black-yellowish color pattern of elytra usually with moderately prevailing yellow; (5) surface of elytra and metaventral disc matt, reticulate; (6) smaller aedeagus with sides of penis subparallel along basal half and gradually tapering in apical half; parameres long, nearly reaching tip of penis with apices truncate; (7) ovipositor shorter, with fewer conspicuous peg-like sensilla; stylus longer compared to length of distal portion of coxite; longitudinal baculum of paraprocts ca 1.6× as long as coxite.

*Ancyronyx helgeschneideri* Freitag & Jäch from Palawan (Philippines) is also quite similar, but differs from the new species in: (1) distinctly smaller size: body length ≤2.0 mm and elytral width up to 0.9 mm; (2) elongate-oval body form; (3) bicolored head and pronotum; (4) elytral color pattern with moderately prevailing dark brown; (5) elytral surface rugulose; (6) distinctly smaller aedeagus with conically tapering penis; parameres long, nearly reaching tip of penis with obliquely truncate apices; (7) ovipositor shorter, stylus and distal portion of coxite relatively longer; longitudinal baculum of paraprocts ca 1.3× as long as coxite.

##### Description of holotype male.

Body form obovate, with rather typical, narrowed “flightless” appearance; elytra strongly convex dorsally, with the highest point at anterior 0.43; BL: 2.97 mm, EW: 1.37 mm, BL/EW: 2.17.

**Figure 3.**
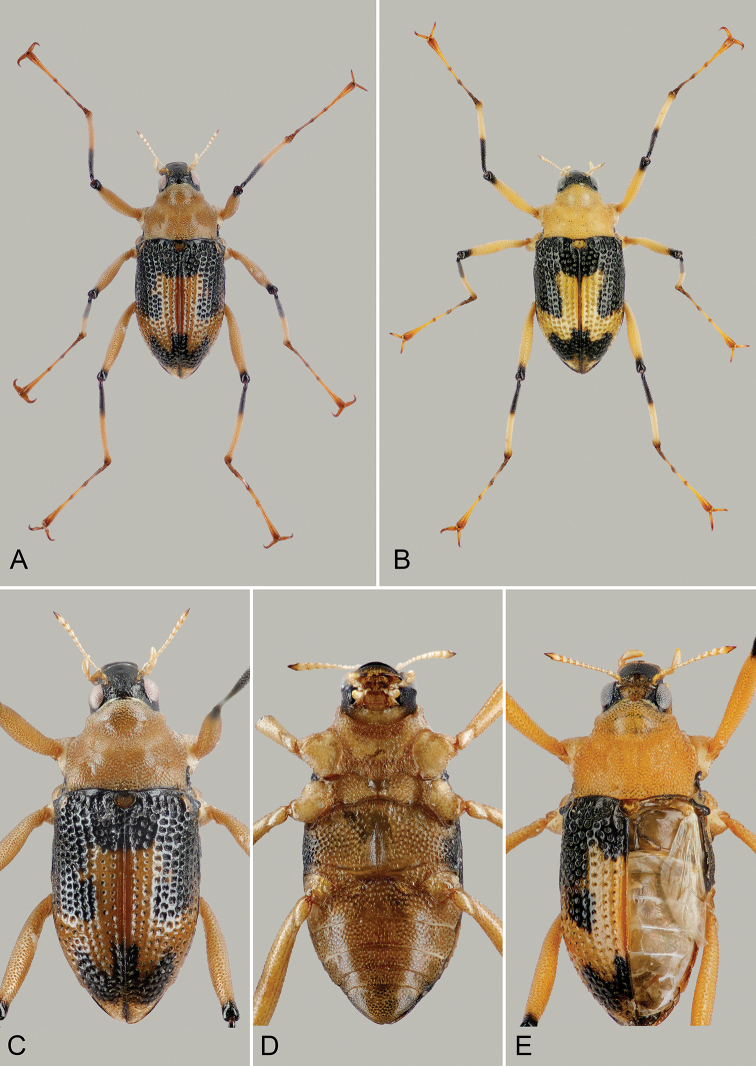
*Ancyronyx lianlabangorum* sp. nov. **A** holotype, dorsal view, body length 2.97 mm **B** female paratype, dorsal view, body length 2.97 mm **C** holotype, dorsal view of body **D** holotype, ventral view of body **E** brachypterous paratype with removed elytron, dorsal view, body length 2.98 mm.

***Coloration* (Fig. 3A).** Labrum black with brownish anterior margin; mandibles dark brown, remaining mouth parts and antennae yellow, except apically darkened terminal antennomere; cranium black dorsally, yellow ventrally; pronotum yellowish; scutellum brownish. Elytra bicolored; anterior half dominantly black with small yellowish spot on humeri and a large central yellowish spot reaching laterally up to the third stria and from anterior fourth up to slightly behind mid-length of the elytra; central spot extending laterad up to margins and posteriad toward apices; thus, the black preapical mark is anchor-shaped. All anepisterna, epimera and lateral portions of metaventrite black; coxae yellow; femora yellowish except small black apical portion; tibiae dark in proximal 0.4 and near articulation with tarsi, yellowish in distal portions; tarsomeres 1–2 darker, tarsomeres 3–5, and claws yellowish brown.

**Figure 4.**
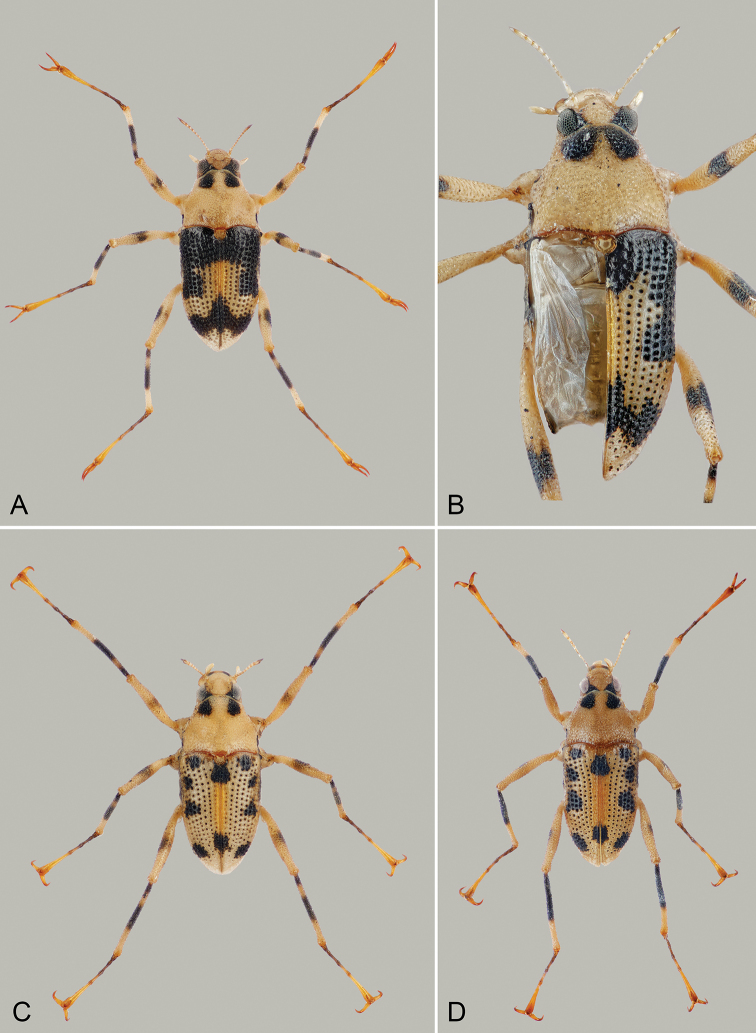
*Ancyronyx pulcherrimus***A** male from Gunung Mulu NP, body length 1.82 mm **B** brachypterous male from Gunung Mulu NP, dorsal view (left elytron and apical segments of abdomen removed), body length 1.92 mm **C** male from Bayur River, dorsal view, body length 1.70 mm **D** male [JK106] from Bayur River, dorsal view, body length 1.70 mm.

***Head*.** Labrum about as long as clypeus, anterior margin slightly concave, almost straight; bipunctate: larger punctures deeper and with fine setae, smaller punctures dense and very shallow, thus surface appearing microreticulate (visible at a magnification of 160×). Clypeus wide, extremely finely punctate-reticulate on anterior half, smooth and shiny on posterior half. Frons and vertex irregularly punctate, punctation more distinct on central portion, punctures intermixed with flat shiny tubercles; frontoclypeal suture almost straight, extremely finely impressed; surface deeply impressed around antennal insertions. Eyes large, subellipsoidal in lateral view, strongly protruding near middle; ratio of horizontal/vertical eye diameter: 1.36. Antennae 11-segmented, about as long as pronotum; each antennomere with a few scattered trichoid setae; antennomeres 9–11 each with two clusters of peg-like setae near distal margins; terminal segment with additional different sensilla. Ratio of length of antennomeres 1–11: 0.09: 0.09: 0.07: 0.05: 0.05: 0.05: 0.05: 0.07: 0.07: 0.07: 0.17 mm. Gena microsculptured; gula narrow, smooth; gular sutures very fine, but discernible; posterior tentorial pits deep and large. HW: 0.66 mm; ID: 0.38 mm.

***Thorax*.** Pronotum (Fig. 3C) distinctly broader than long, PL/MW: 0.76, PL: 0.87 mm, MW: 1.15 mm, widest near posterior angles; anteriorly attenuate; anterior margin strongly arcuate, bordered by narrow protruding translucent edge; hypomeral portion almost entirely visible in dorsal view; anterior transverse groove distinctly impressed, forming a widely open “V”; area posterior of transverse groove strongly gibbous and distinctly higher than anterior portion; mesal longitudinal carina absent; posterolateral oblique grooves shallow, hardly discernible. Pronotal surface with irregularly arranged, flat shiny, sometimes cordiform tubercles, each of which bears a short seta; tubercles in anterior transverse groove dense and coalescent (forming a nearly rasp-like structure), laterad and posteriad gradually smaller and less densely arranged. Prosternum irregularly densely and roughly punctate, very short in front of procoxae; anterior margin strongly concave, unusually thickened; prosternal process distinctly transverse, about as wide as head, depressed near apex, sides slightly sinuate; posterior margin widely rounded, feebly protruding posteriad. Scutellum subpentagonal, smooth and shiny. Elytra (Fig. 3C) moderately elongate and only somewhat wider than pronotum, EL: 2.10 mm, EW: 1.37 mm; convex in lateral view, with highest point at anterior 0.43; lateral sides visible in dorsal view; lateral outline slightly narrowed at level of metacoxae, then gradually convergent towards conjointly rounded apices; elytral punctures forming ten more or less regular rough rows; seven rows between suture and shoulder including accessory scutellary striole (five punctures); punctures large, round to subquadrate and deeply impressed on disc and laterally, smaller and less distinct posteriorly; interstices and intervals very narrow and moderately raised on disc and laterally, posteriorly becoming wider and flatter; surface shiny with sparse short setae and some longer trichoid setae (partly abrased); humeri small but prominent. Lateral rim visible from above, not serrate, finely carinate, positioned at abdominal recess of laterosternites and laterally produced margin of ventrites, very tightly fitted to abdomen. Mesoventrite approximately half as long as prosternum; mesoventral cavity deep and robust; surface strongly and irregularly punctate; without mesoventral discrimen; posterior angles rounded and moderately protruding, raised and partially concealing mesocoxae; mesepimeron small, protruding laterad into short blunt process visible in dorsal view. Metaventrite (Fig. 3D) along midline about as long as combined length of prosternum and mesoventrite; anterior margin arcuate, anterior portion declined; disc with deep wide longitudinal depression mesally, discrimen finely raised, metakatepisternal suture fine; surface densely irregularly roughly punctate, punctures coarser and denser laterally. Hind wing distinctly shorter than elytron, unfunctional, reduced in length and venation (Fig. 3E). Forelegs very long, about 1.73× as long as body; pro- and mesocoxae large and prominent, strongly protruding laterally at least partially visible in dorsal view, bluntly drop-shaped; metacoxae smaller and less protruding laterally. Femora and tibiae with short setae and characteristic pattern of smooth, shiny elongate flat tubercles; tibiae with a few longer setae; distal tarsomeres with several longer setae near apex; claws large, strongly curved, base with three teeth, two larger ones and a smaller one.

***Abdomen* (Fig. 3D).** Intercoxal process moderately longer than length of first ventrite posterior of metacoxae, anterior margin very wide, arcuate; anteromedial portion on same level with bottom of metaventral depression; rows of deep punctures arranged along anterior margin; ratio of length of ventrites 1–5: 0.37: 0.23: 0.23: 0.18: 0.30 mm. Surface of ventrites 2–5 with sparse punctures and flat setiferous, almost cordiform tubercles; punctures more distinct on medial area; tubercles more prominent and more conspicuous laterally; fifth ventrite densely tuberculate.

Sternite IX ca 600 μm long and very robust (Fig. 7B); apical margin moderately arcuately emarginate; lateroapical portion with a few moderately long setae and numerous microtrichia; paraprocts not reaching beyond apical margin; ventral strut short and wide. Tergite VIII with complete transverse sinuate ridge dividing anterior and posterior portion, anterior surface with dense microtrichia; sides subparallel along basal half and arcuate in apical half; surface with sparse hair-like setae, which are more robust and longer on sublateral portions than along lateral margins.

***Aedeagus*** (Fig. 6A–C) ca 600 μm long; penis including lateral basal apophyses ca 2.4× as long as phallobase, robust and well sclerotized, sides subparallel, short apical portion abruptly narrowed and moderately curved ventrad (lateral view), dorsolateral portion with numerous shorter setae; apex widely rounded; basal apophyses long; ventral sac large; fibula conspicuous and long, moderately wide; dorsal portion mesally with unusual, more distinctly pigmented/sclerotized longitudinal structure (similar to fibula); surface of endophallus with spinules; corona distinct. Phallobase asymmetrical. Parameres robust and wide, reaching apical third of penis, broadest near base, narrowest near middle; dorsal and ventral outline moderately concave; apices wide, rounded and emarginated in middle, appearing nearly double-peaked (in dorsal/ventral view); apical and lateral surface of parameres with short setae.

##### Female abdomen and ovipositor.

Tergite VIII with strong complete transverse sinuate ridge; anterior portion with dense microtrichia; posterior portion more distinctly pigmented, with two sublateral clusters of strong conspicuous hair-like setae; lateral sides arcuate; apical margin with protruding translucent edge. Sternite VIII robust, median strut short and wide, apex moderately emarginated with more robust hair-like setae (Fig. 7C). Ovipositor (Fig. 7A) 590 μm long; stylus narrow and almost straight, relatively short: ca 0.43× as long as distal portion of coxite. Coxite robust, short and wide, rounded at posterolateral angle; distal portion ca 1.16× as long as wide at middle, slightly bent, with numerous conspicuous stout peg-like setae and with a few thinner peg-like setae, the latter mainly at apical portion; inner margin densely pubescent; proximal portion about as long as distal portion, with several types of peg-like and short fine hair-like setae (omitted in Fig. 7A). Transverse baculum well sclerotized; longitudinal baculum of paraprocts (valvifers) almost twice as long as coxite (measured from the apical margin of coxite to point where it is joining the transverse baculum).

##### Secondary sexual dimorphism.

Females are on average larger and broader than males from the same population, with more elongate ventrite 5, and the longitudinal depression of their metaventrite is broader but shallower.

##### Variability.

The specimens vary moderately in size (Table 4). In contrast to, e.g., *Ancyronyx sarawacensis* and *A. procerus*, the elytral color pattern varies only slightly (Fig. 3B). Only a single specimen possesses a bicolored head (Fig. 3E). The surface structure of the head varies moderately in the density of the tubercles and punctures, and most of the specimens lack any trace of the frontoclypeal suture. The shape of the elytra indicates that the specimens have reduced hind wings, in contrast to the apterous 32 specimens of *A. variegatus* examined by Shepard (2019).

##### Habitat.

At the type locality (Pa’ Ramudu River), the specimens were sampled from an approximately 200 m long stretch. The river was shallow, moderately meandering, about 7–15 m wide, slowly flowing through a degraded primary forest (data for 5 March 2019). Shallow reaches (10–40 cm) with slow current alternated with deeper pools (50–120 cm); the river was partly shaded by the riparian vegetation including large old trees, dense bamboo groves, shrubs and massive tree ferns (*Cyathea* sp.). The substrate contained gravel, sand, stones and exposed boulders; however, some shallow sections showed bedrock ledge only. Submerged wood, as well as large packs of bamboo roots were present mainly 50–100 m downstream of the connection with the Pa’ Masia River. *Ancyronyx lianlabangorum* sp. nov. was collected exclusively from massive, submerged logs in deeper pools with slow current. In contrast, submerged bamboo rootlets and smaller pieces of wood were inhabited only by *Ancyronyx sarawacensis* and *A. acaroides*.

**Figure 5.**
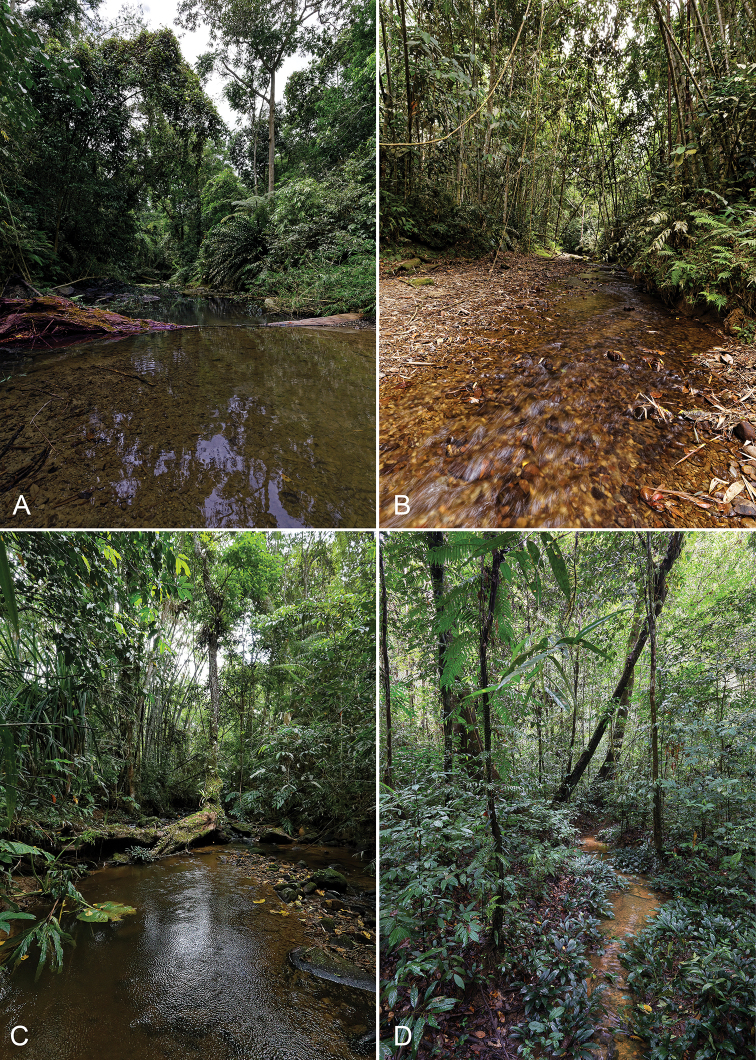
Habitats of *Ancyronyx lianlabangorum* sp. nov. (**A–C**) and *A. pulcherrimus* (**D**) **A** type locality, Pa’ Ramudu River **B** Pa’ Masia River **C** Pa’ Kasi River **D** shaded shallow creek in primary forest, Gunung Mulu NP.

The Pa’ Masia River represents a headwater stream in degraded primary forest (Fig. 5B). During our sampling on 6 March 2019, it was about 5–50 cm deep, 3–7 m wide and slowly flowing. Adults inhabited submerged wood in several deeper pools with almost no current.

A single female sampled on 28 June 2018 was found on a large submerged log in a pool of the entirely shaded, shallow Pa’ Kasi near the connection with Pa’ Kelapang River in the vicinity of Kampung Ramudu (Fig. 5C). The river bottom contained mainly firmly arranged pebbles and rocks.

The altitude of the collection sites ranges from 900 to 970 m a.s.l.

Neither the intensive collection efforts in Pa’ Kelapang River near connections with Pa’ Ramudu, Pa’ Kasi, Pa’ Ngaruren and Pa’ Buah rivers near Kampung Ramudu revealed any further adults or larvae of *A. lianlabangorum* sp. nov., nor the samplings of the upstream sections of Pa’ Kelapang River near Batu Patong, Pa’ Mada as well as Pa’ Umor. However, in all these habitats we found submerged autochthonous wood inhabited by *Ancyronyx sarawacensis*, *A. procerus*, *A. acaroides*, and several species of *Graphelmis*, *Leptelmis* Sharp, *Elmomorphus* Sharp and *Stenomystax* Kodada, Jäch & Čiampor.

##### Distribution.

This species is known from a few small, slowly flowing tributaries of Pa’ Kelapang River near Kampung Ramudu, Kelabit Highlands, Sarawak.

##### Etymology.

We named the species in honor of David Lian Labang (“Uncle David”) and his son David Lian Labang Jr from Bareo (Kelabit Highlands, Sarawak). They run Labang Longhouse Lodge in Bareo. Ján Kodada and David Selnekovič have always been happy to take advantage of this fabulous accommodation during several field trips. Uncle David is a retired, well-known employee of the Forest Department Sarawak, who contributed to the knowledge and conservation of Sarawak’s nature. We are most grateful to both for many stories and shared information about the terrain, nature, culture, as well as for their endless repertoire of jokes!

**Figure 6.**
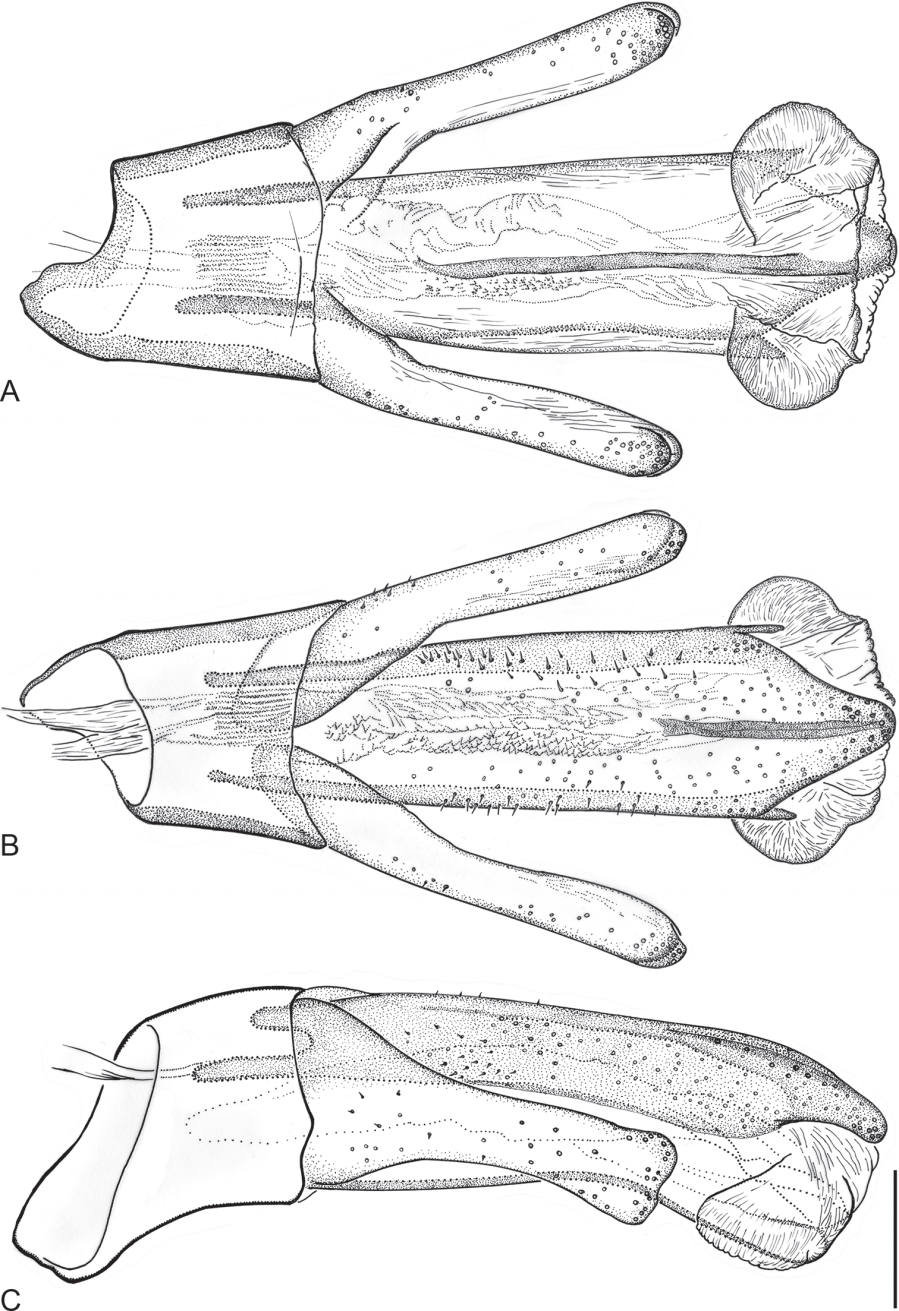
*Ancyronyx lianlabangorum* sp. nov., aedeagus of holotype **A** ventral view **B** dorsal view **C** lateral view. Scale bar: 0.1 mm.

**Figure 7.**
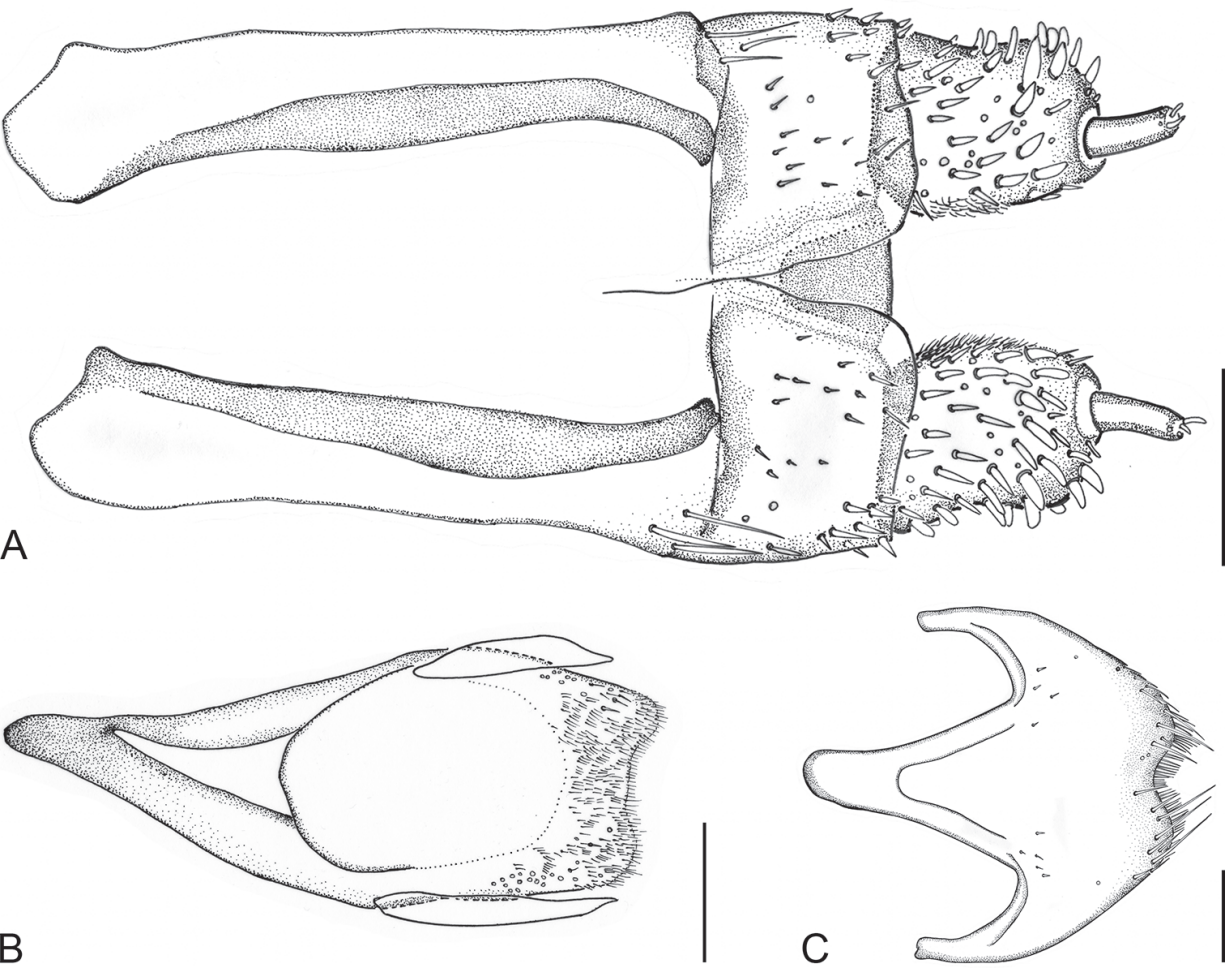
*Ancyronyx lianlabangorum* sp. nov. **A** ovipositor, paratype [JK145], ventral view **B** male sternite IX, holotype, ventral view **C** female sternite VIII, paratype [JK145], ventral view. Scale bars: 0.1 mm.

#### Ancyronyx pulcherrimus

Taxon classificationAnimaliaColeopteraElmidae

Kodada, Jäch & Čiampor, 2014

ACEF8DE1-61EA-5B97-9C88-CBF774952C80

##### Type locality.

Ingei River, forest pool, , Belait District, Brunei.

##### Material examined.

**Type material**. ***Holotype*** ♂ (BM): “Brunei: Belait Distr. Sg. Ingei, forest pool near Base Camp, 14.VI.2010 leg. Mayyer Ling (34)”. ***Paratypes*** (NMW, UB): 1 ♂ and 2 ♀♀, same label data as holotype.

##### Additional material

(CCB, CFDS, CKB, NMW, UBDM).

**Lineage 1. Sarawak** 9 ♂♂ [incl. JK100, JK213, JK214, JK215], 15 ♀♀ [incl. JK101, JK102, JK208, FZ1632]: “Malaysia, Sarawak, Marudi distr., Gunung Mulu NP, 16.10.2018, (40) 04.0267°N, 114.8234°E, 55 m a.s.l., small stream in primary forest, Kodada & Selnekovič lgt.”. **Brunei** 2 ♂♂, 1 ♀ [FR324]: “Brunei: Temburong; small Sungai Peradayan; rivulet, subm. wood; dist. primary forest; c. 25m a.s.l. c. 04.11.1997 leg. Mendoza (6f)”.

**Lineages 2 and 3. Sarawak** 5 ♂♂ [incl. JK105, JK106], 1 ♀ [JK12]: “Malaysia, Sarawak, Kuching distr., Bayur riv. near Kampung Bayur, 20.10.2018, 1°14'42.3"N, 110°17'35.3"E 40 m a.s.l., J. Kodada & D. Selnekovič lgt.”; 2 ♂♂ [JK197, JK199], 4 ♀♀ [incl. JK193, JK195]: same locality and collectors, but 22.02.2019, (No.43); 1 ♂ [FR344]: “Malaysia: Sarawak, Kg. Bayur 35km SSE Kuching, small hill river Bayur R.; run, subm. wood; second. veget./farmland; c. 50m a.s.l., c. 12.11.1993 leg. Mendoza (19f)”.

##### Variability.

*Ancyronyx pulcherrimus* was represented by three barcode lineages, which are tentatively considered conspecific in the absence of further evidence. The specimens of the first lineage were collected in northern Sarawak and Brunei, while the specimens of the second and third lineages occur sympatrically in southwestern Sarawak. The first lineage is characterized by: (1) elytra predominantly black with a central yellowish, nearly X-shaped pattern and yellowish apices; (2) femora with black band between middle and proximal fourth; and (3) tibiae black on proximal three-quarters (compare Fig. 4 in Kodada et al. 2014). The type specimens are rather uniform in these characters, and all individuals recently collected in Gunung Mulu NP generally agree with them. However, there is some minor variation in the extent of the black coloration of the elytra, pronotum and legs (Fig. 4A, B). The examination of the hind wings of six specimens of the Gunung Mulu population and three specimens from Temburong (Brunei) revealed only short wings not reaching the abdominal apex (Fig. 4B). Macropterous or apterous forms were not detected, and the elytral shape of the remaining specimens suggests that they are flightless too.

The specimens of the second and third lineages differ from the type specimens in the predominantly yellowish elytra, which are provided with several black lateral and sutural spots of variable size. The pronotal spots are usually smaller, and the dark femoral bands are smaller, paler or obsolete (Fig. 4C, D). The examination of their hind wings revealed one macropterous and five brachypterous specimens.

Lineages detected by the analysis of COI nucleotide sequences were not confirmed by PCA analyses of morphometric characters (Fig. 2, Table 4) or corroborated by the genital morphology.

##### Habitat and syntopic taxa.

The stream inhabited by a large population of *A. pulcherrimus* was a small, slowly flowing, very shallow, and entirely shaded meandering creek in Gunung Mulu NP (55 m a.s.l.). It included large amounts of accumulated leaves, and the bottom substrate contained sand and gravel, as well as woody debris (Fig. 5D). The specimens co-occurred with *Ancyronyx sarawacensis* and two new species of Elmidae (*Okalia* Kodada & Čiampor and *Leptelmis*).

The second population was found in a moderately wide (5–8 m) lowland river near Kampung Bayur (Kuching Division); the sampled stretch was shallow and meandering, with sandy/gravelly substrate containing submerged logs, woody debris, and leaves. All specimens were collected exclusively in reaches with stronger current from submerged logs together with *Ancyronyx procerus*, *A. acaroides*, and several other Elmidae (*Leptelmis* sp. and *Graphelmis* spp. of the *G. picta* (Reitter) and *G. marshalli* Hinton species groups).

##### Distribution.

*Ancyronyx pulcherrimus* was so far known only from Brunei (Jäch et al. 2016). The species is here recorded for the first time from Malaysia, where it was collected in Gunung Mulu NP (northern Sarawak) and in the Kuching Division (southwestern Sarawak).

Our recent collecting activities found *A. pulcherrimus* to be less abundant and less widely distributed than *A. sarawacensis*, *A. procerus*, or *A. acaroides*, probably also due to the low dispersal ability of flightless populations.

## Discussion

Striking and specific yellowish-black color patterns usually characterize *Ancyronyx* adults (e.g., Jäch 1994, 2004; Kodada et al. 2014). However, the analysis of COI sequences confirmed the existence of intraspecific variability regarding coloration and other morphological characters within populations of *A. sarawacensis* and *A. procerus* (Kodada et al. 2020). All eight *Ancyronyx* species known from Borneo live on submerged wood (woody debris) in running water. They mainly occur in primary or slightly degraded forests, although some specimens were sampled in secondary forests or forest remnants as well. Not any specimen was found in large, polluted rivers or at light traps placed near such rivers in the Kuching Division during our sampling in 2018 and 2019 (Kodada et al. 2020). *Ancyronyx lianlabangorum* sp. nov. and *A. pulcherrimus* represent two obviously rare, flightless species with low dispersal ability. Massive deforestation generally threatens flightless species, which may survive mainly in protected, undisturbed regions. Apparent changes in seasonal patterns affecting precipitation and the intensity and frequency of extreme weather events will strongly impact the populations of these two species.

Specimens of *Ancyronyx lianlabangorum* sp. nov. were sampled from a morphologically uniform, small population, and also their nucleotide COI sequences showed minimal divergences (Suppl. material 1: Table S1).

Surprisingly, the nucleotide COI divergences among the three lineages of *A. pulcherrimus* showed taxonomic complexity (Suppl. material 2: Table S2). To decide whether they represent one, two, or even three species is challenging and not yet resolved, as there are no standardized procedures to address the taxonomic status of such genetic lineages. However, all specimens were grouped to a single, well-supported but unstructured clade in the tree inferred from amino acid COI sequences (Suppl. material 3: Fig. S1), as all their pairwise distances dropped to 0.00% (Suppl. material 4: Table S3). The very uniform genital and external morphology also supports the homogeneity of the *A. pulcherrimus* clade. Consequently, the differences in the color pattern are considered here as a result of intraspecific variability as reported for other Bornean and Philippine *Ancyronyx* (Kodada et al. 2020; SabColeoptera et al. in press).

Additionally, the nucleotide sequence divergences among other well-defined *Ancyronyx* species from Borneo were considerably higher, ranging from 9.4% to 20.0% (Table 2). They correspond to unusually deep interspecific divergences detected in a regional survey of 1872 northern European species of Coleoptera as well (Pentisaari et al. 2014). The effectiveness of DNA barcodes in the identification of Coleoptera varies. While barcoding worked quite well when tested for thousands of European beetles (Pentisaari et al. 2014; Hendrich et al. 2015), problems for evolutionary young and radiating species have also been reported (e.g., Hendrich et al. 2010; Balke et al. 2013; Komarek and Freitag 2020). The higher diversification within the *A. pulcherrimus* clade agrees with a study of Silphidae from Japan, including a meta-analysis of 51 species from 15 families, which revealed a higher genetic differentiation and a higher diversification rate among populations in the flightless lineages than in the flight-capable lineages (Ikeda et al. 2012). Thus, the possibility of the existence of multiple distinct species within the *A. pulcherrimus* clade cannot be ruled out.

Final solutions for the taxonomic status of the three *A. pulcherrimus* lineages require a larger sample size, comprising more localities from a more extensive distributional range. Such an approach should also be combined with the use of nuclear markers.

## Supplementary Material

XML Treatment for Ancyronyx lianlabangorum

XML Treatment for Ancyronyx pulcherrimus

## Supplementary materials

Supplementary material 1 Table S1. Pairwise genetic distances (Kimura 2-parameter distance) between nucleotide sequences of *Ancyronyx* and *Graphelmis* species based on the 648 bp barcoding fragment of COI. Data type DNA data table https://binary.pensoft.net/file/486559This dataset is made available under the Open Database License (http://opendatacommons.org/licenses/odbl/1.0/). The Open Database License (ODbL) is a license agreement intended to allow users to freely share, modify, and use this Dataset while maintaining this same freedom for others, provided that the original source and author(s) are credited.Ján Kodada, Manfred A. Jäch, Hendrik Freitag, Zuzana Čiamporová-Zaťovičová, Katarína Goffová, Dávid Selnekovič, Fedor Čiampor Jr

Supplementary material 2 Table S2. DNA barcoding patterns of the variable nucleotide positions in the 648 bp long COI sequence alignment of the *Ancyronyx pulcherrimus* clade. Data type DNA data table https://binary.pensoft.net/file/486560This dataset is made available under the Open Database License (http://opendatacommons.org/licenses/odbl/1.0/). The Open Database License (ODbL) is a license agreement intended to allow users to freely share, modify, and use this Dataset while maintaining this same freedom for others, provided that the original source and author(s) are credited.Ján Kodada, Manfred A. Jäch, Hendrik Freitag, Zuzana Čiamporová-Zaťovičová, Katarína Goffová, Dávid Selnekovič, Fedor Čiampor Jr

Supplementary material 3 Figure S1. Maximum Likelihood tree inferred from aligned COI mtDNA amino acids sequences. Data type image https://binary.pensoft.net/file/486561This dataset is made available under the Open Database License (http://opendatacommons.org/licenses/odbl/1.0/). The Open Database License (ODbL) is a license agreement intended to allow users to freely share, modify, and use this Dataset while maintaining this same freedom for others, provided that the original source and author(s) are credited.Ján Kodada, Manfred A. Jäch, Hendrik Freitag, Zuzana Čiamporová-Zaťovičová, Katarína Goffová, Dávid Selnekovič, Fedor Čiampor Jr

Supplementary material 4 Table S3. Pairwise genetic distances (p-distance) between 29 amino acid sequences (216 positions) of *Ancyronyx* species and the genus *Graphelmis* (outgroup). Data type DNA data table https://binary.pensoft.net/file/486562This dataset is made available under the Open Database License (http://opendatacommons.org/licenses/odbl/1.0/). The Open Database License (ODbL) is a license agreement intended to allow users to freely share, modify, and use this Dataset while maintaining this same freedom for others, provided that the original source and author(s) are credited.Ján Kodada, Manfred A. Jäch, Hendrik Freitag, Zuzana Čiamporová-Zaťovičová, Katarína Goffová, Dávid Selnekovič, Fedor Čiampor Jr
