# Supplementary material for: Ancyronyx lianlabangorum sp. nov., a new spider riffle beetle from Sarawak, and new distribution records for A. pulcherrimus Kodada, Jäch & Čiampor based on DNA barcodes (Coleoptera, Elmidae)
Source: Zookeys. 2020 Dec 14;1003:31–55. doi: 10.3897/zookeys.1003.55541 (PMC7752891; doi:10.3897/zookeys.1003.55541)
Supplement: Supplementary material 2 — Table S2. DNA barcoding patterns of the variable nucleotide positions in the 648 bp long COI sequence alignment of the Ancyronyx pulcherrimus clade. [file zookeys-1003-031-s002.docx]

**Table S2.** DNA barcoding patterns of the variable nucleotide positions in the 648 bp long COI sequence alignment of the *Ancyronyx pulcherrimus* clade.

| **Position in alignmet** | | 14 | 35 | 107 | 128 | 152 | 161 | 200 | 230 | 263 | 270 | 320 | 332 | 338 | 339 | 344 | 347 | 359 | 380 | 389 | 392 | 401 | 431 | 446 | 455 | 458 | 476 | 488 | 563 | 599 | 617 | 620 | 632 | 638 |
| --- | --- | --- | --- | --- | --- | --- | --- | --- | --- | --- | --- | --- | --- | --- | --- | --- | --- | --- | --- | --- | --- | --- | --- | --- | --- | --- | --- | --- | --- | --- | --- | --- | --- | --- |
| ***Ancyronyx pulcherrimus* TYPE MFH** | | **A** | **A** | **C** | **C** | **A** | **T** | **G** | **C** | **C** | **C** | **A** | **C** | **T** | **C** | **T** | **C** | **T** | **G** | **T** | **A** | **T** | **A** | **A** | **C** | **G** | **C** | **A** | **T** | **T** | **A** | **G** | **C** | **C** |
| **Lineage 1** | *Ancyronyx pulcherrimus* FZ1632A | **G** | **.** | **.** | **.** | **.** | **.** | **.** | **.** | **.** | **.** | **.** | **.** | **.** | **.** | **.** | **.** | **.** | **.** | **.** | **.** | **.** | **.** | **.** | **.** | **.** | **.** | **.** | **.** | **.** | **.** | **.** | **.** | **.** |
|  | *Ancyronyx pulcherrimus* FR324 | . | **.** | **.** | **.** | **.** | **.** | **.** | **.** | **.** | **.** | **.** | **.** | **.** | **.** | **.** | **.** | **.** | **.** | **.** | **.** | **.** | **.** | **.** | **.** | **.** | **.** | **.** | **.** | **.** | **.** | **.** | **.** | **.** |
|  | *Ancyronyx pulcherrimus* JK100 | . | **.** | **.** | **.** | **.** | **.** | **.** | **.** | **.** | **.** | **.** | **.** | **.** | **.** | **.** | **.** | **.** | **.** | **.** | **.** | **.** | **.** | **.** | **.** | **.** | **.** | **.** | **.** | **.** | **.** | **.** | **.** | **.** |
|  | *Ancyronyx pulcherrimus* JK101 | . | **.** | **.** | **.** | **.** | **.** | **.** | **.** | **.** | **.** | **.** | **.** | **.** | **.** | **.** | **.** | **.** | **.** | **.** | **.** | **.** | **.** | **.** | **.** | **.** | **.** | **.** | **.** | **.** | **.** | **.** | **.** | **.** |
|  | *Ancyronyx pulcherrimus* JK102 | . | **.** | **.** | **.** | **.** | **.** | **.** | **.** | **.** | **.** | **.** | **.** | **.** | **.** | **.** | **.** | **.** | **.** | **.** | **.** | **.** | **.** | **.** | **.** | **.** | **.** | **.** | **.** | **.** | **.** | **.** | **.** | **.** |
|  | *Ancyronyx pulcherrimus* JK214 | . | **.** | **.** | **.** | **.** | **.** | **.** | **.** | **.** | **.** | **G** | **.** | **.** | **.** | **.** | **.** | **.** | **.** | **.** | **.** | **.** | **.** | **.** | **.** | **.** | **.** | **.** | **.** | **.** | **.** | **.** | **.** | **.** |
|  | *Ancyronyx pulcherrimus* JK215 | . | **.** | **.** | **.** | **.** | **C** | **.** | **.** | **.** | **.** | **.** | **.** | **.** | **.** | **.** | **.** | **.** | **.** | **.** | **.** | **.** | **.** | **.** | **.** | **.** | **.** | **.** | **.** | **.** | **.** | **.** | **.** | **.** |
|  | *Ancyronyx pulcherrimus* JK213 | . | **.** | **.** | **.** | **.** | **C** | **.** | **.** | **.** | **.** | **.** | **.** | **.** | **.** | **.** | **.** | **.** | **.** | **.** | **.** | **.** | **.** | **.** | **.** | **.** | **.** | **.** | **.** | **.** | **.** | **.** | **.** | **.** |
|  | *Ancyronyx pulcherrimus* JK208 | . | **.** | **.** | **.** | **.** | **.** | **.** | **.** | **.** | **.** | **.** | **.** | **.** | **.** | **.** | **.** | **.** | **.** | **.** | **.** | **.** | **.** | **.** | **.** | **.** | **.** | **G** | **.** | **.** | **.** | **.** | **.** | **.** |
| **Lineage 2** | *Ancyronyx pulcherrimus* JK195 | **.** | **.** | **.** | **.** | **.** | **.** | **A** | **T** | **.** | **.** | **.** | **.** | **.** | **T** | **.** | **.** | **.** | **.** | **C** | **G** | **.** | **.** | **.** | **.** | **A** | **.** | **G** | **C** | **C** | **.** | **A** | **T** | **T** |
|  | *Ancyronyx pulcherrimus* JK106 | **.** | **.** | **.** | **T** | **.** | **.** | **A** | **T** | **.** | **.** | **.** | **.** | **.** | **T** | **.** | **.** | **.** | **.** | **C** | **G** | **.** | **.** | **.** | **.** | **A** | **T** | **G** | **C** | **C** | **.** | **A** | **T** | **T** |
|  | *Ancyronyx pulcherrimus* JK193 | **.** | **.** | **.** | **T** | **.** | **.** | **A** | **T** | **.** | **.** | **.** | **.** | **.** | **T** | **.** | **.** | **.** | **.** | **C** | **G** | **.** | **.** | **.** | **.** | **A** | **T** | **G** | **C** | **C** | **.** | **A** | **T** | **T** |
| **Lineage 3** | *Ancyronyx pulcherrimus* FR344 | **.** | **.** | **T** | **.** | **G** | **.** | **.** | **T** | **T** | **T** | **.** | **T** | **.** | **.** | **C** | **.** | **.** | **A** | **C** | **.** | **C** | **.** | **.** | **T** | **A** | **.** | **.** | **.** | **C** | **G** | **A** | **T** | **.** |
|  | *Ancyronyx pulcherrimus* JK12 | **.** | **.** | **T** | **.** | **G** | **.** | **.** | **T** | **T** | **T** | **.** | **T** | **.** | **.** | **C** | **T** | **.** | **A** | **C** | **.** | **C** | **.** | **G** | **T** | **A** | **.** | **.** | **.** | **C** | **.** | **A** | **T** | **.** |
|  | *Ancyronyx pulcherrimus* JK199 | **.** | **.** | **T** | **.** | **G** | **.** | **.** | **T** | **T** | **T** | **.** | **T** | **.** | **.** | **C** | **T** | **.** | **A** | **C** | **.** | **C** | **T** | **G** | **T** | **A** | **.** | **.** | **.** | **C** | **G** | **A** | **T** | **.** |
|  | *Ancyronyx pulcherrimus* JK105 | **.** | **.** | **T** | **.** | **G** | **.** | **.** | **T** | **T** | **T** | **.** | **T** | **C** | **.** | **C** | **T** | **C** | **A** | **C** | **.** | **C** | **.** | **G** | **T** | **A** | **.** | **.** | **.** | **C** | **G** | **.** | **T** | **.** |
|  | *Ancyronyx pulcherrimus* JK197 | **.** | **G** | **T** | **.** | **G** | **.** | **.** | **T** | **T** | **T** | **.** | **T** | **C** | **.** | **C** | **T** | **.** | **A** | **C** | **.** | **C** | **.** | **G** | **T** | **A** | **.** | **.** | **.** | **C** | **.** | **A** | **T** | **.** |

The first line sequence represents a most frequent haplotype **(TYPE MFH)** obtained from the specimens morphologically similar to the **type specimens** and sampled ca15 km from the type locality. Dots (.) indicate sequence identity to the reference sequence on the first line. The first lineage involves sequences of specimens from Gunung Mulu National Park (Sarawak) and of one specimen from Brunei (FR324). The dataset shows only a minimal nucleotide substitution pattern within the lineage and morphological characters correspond to those of the type specimens of *A. pulcherrimus*. The second and the third lineages represent specimens sampled ca 600 km from the type locality, both are morphologically similar to each other, but they differ from the first lineage in the elytral color pattern. The second and third lineages are sympatric, but they differ in the pattern of nucleotide substitutions. However, there were no morphological/metric characters in the specimens sequenced found that allow to distinguish these lineages. Specimen JK197 is a macropterous **♂** and specimens JK195 (♀), JK199 (♂) and JK214 (♂) are all brachypterous.
