# Supplementary figures and images for: Ancyronyx lianlabangorum sp. nov., a new spider riffle beetle from Sarawak, and new distribution records for A. pulcherrimus Kodada, Jäch & Čiampor based on DNA barcodes (Coleoptera, Elmidae)
Source: Zookeys. 2020 Dec 14;1003:31–55. doi: 10.3897/zookeys.1003.55541 (PMC7752891; doi:10.3897/zookeys.1003.55541)

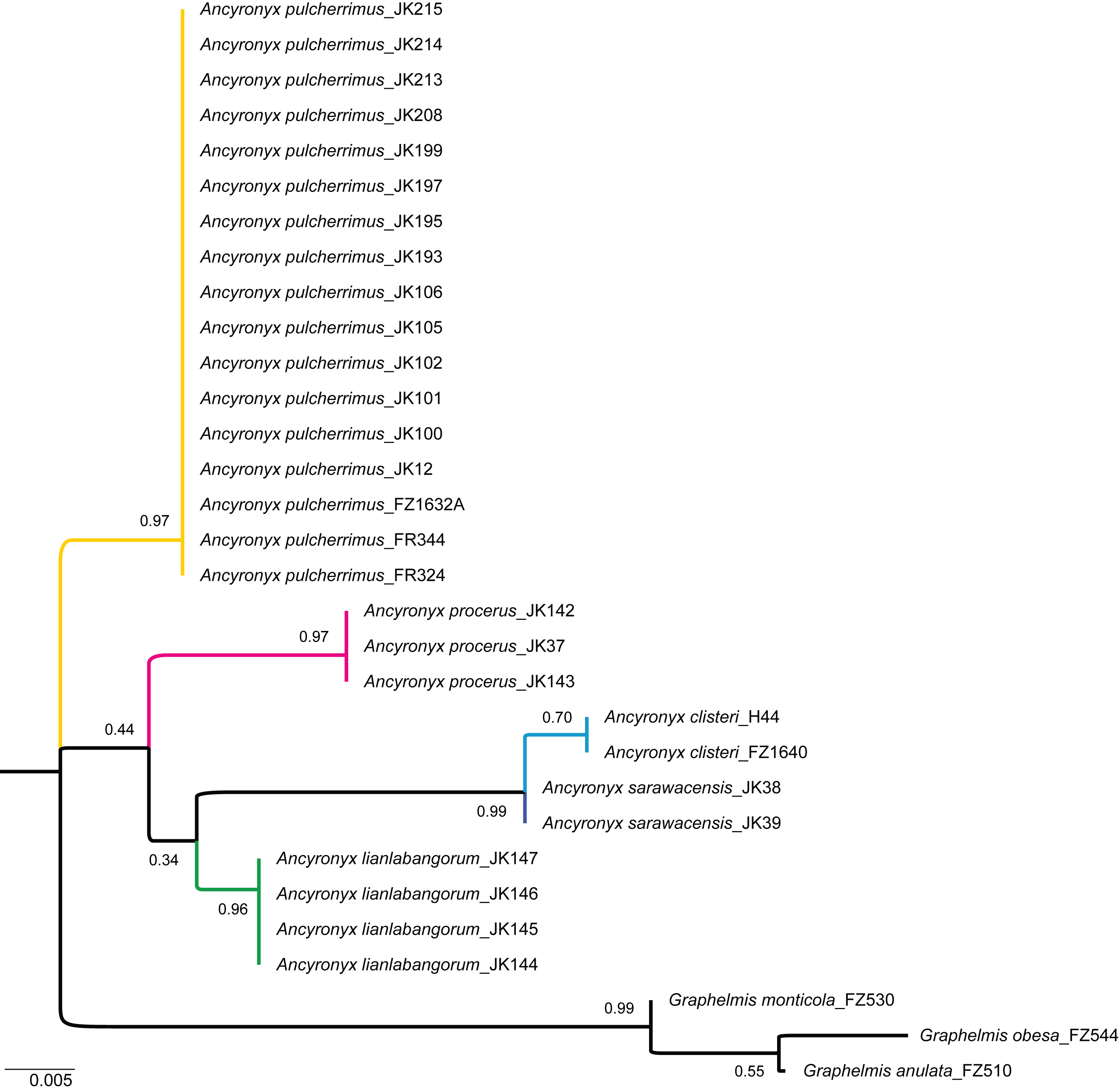

Supplement: Supplementary material 3 — Figure S1. Maximum Likelihood tree inferred from aligned COI mtDNA amino acids sequences. [file zookeys-1003-031-s003.tif]
